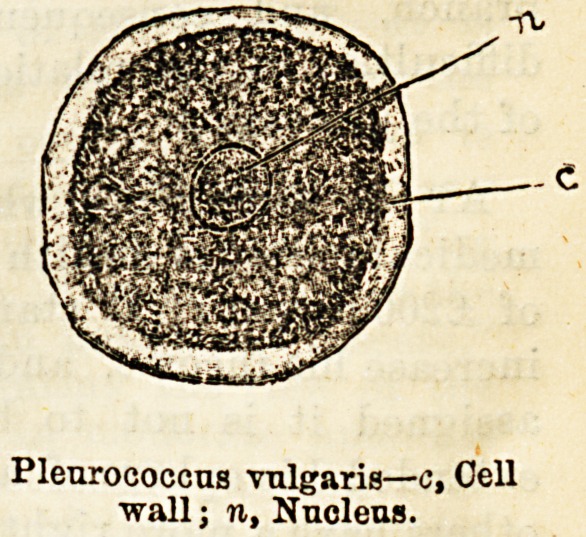# On the Cellular Structure of Living Organisms.—II

**Published:** 1893-11-04

**Authors:** 


### On the Cellular Structure of Living Organisms.—II.

The transition from the non-cellular to the cellular condition of organic structure is by no means so abrupt as might, perhaps, be supposed. For if a myxomycete, such as that mentioned in our last article under the name of "Flowers of Tan," be followed through its entire life, there will be seen stages in which it actually does exhibit a very definite cell-form. After passing, it may be, a long term of existence in the form of a creeping plasmodium, the time will arrive when it will change its character, and the entire mass will break up into small protoplasmic particles, each of which contains a single nucleus; and this nucleated fragment then becomes invested by a cell wall, and forms thus an isolated body, often spoken of as a spore. The details of spore formation vary a great deal in the different genera of which the class Myxomycetes is composed, but they all possess the important property in common of being able to resist the action of unfavourable surroundings, owing to their protoplasm existing in a dormant condition within the envelope or spore-membrane. It is interesting to trace the further fate of these spores, when they proceed to develop or *germinate*. This process takes place under favourable conditions of warmth and moisture; the protoplasm ruptures the spore-membrane, and issues from it as a motile body which propels itself through the water by means of a whip-like filament, resembling a much attenuated and elongated pseudopodium, and, like this structure, is of protoplasmic nature. It is often spoken of as a *flagellum*. In the substance of the moving mass, besides a nucleus, there may often be distinguished a circular spot which alternately appears and disappears. This structure, perhaps, represents a very rudimentary form of excretory organ, and is termed the contractile vacuole. When full it contains a watery liquid, and the disappearance is due to the active contraction of the protoplasm surrounding the vacuole, whereby its contents are squeezed out through the organism.

Paramæcium aurelia—C, Cilia; N, Nucleus; CV, Contractile vacuole; F, Water globules containing solid food particles; G, Gullet or mouth.

Eventually, however, these naked flagellate bits of protoplasm become more sluggish in their movements, the flagellum is withdrawn, and further locomotion takes place by means of ordinary pseudopodia. Sometimes, at this stage, the individuals become spherical in shape, and again secrete an enclosing wall, and in this condition the protoplasm is said to be *encysted*. This process of encystment depends largely on external conditions, and eventually the protoplasm again escapes as a naked mass from the bag or cyst. And now a remarkable phenomenon may be observed. A number of originally free individuals congregate and coalesce, and thus a plasmodium is formed, in which the independent units have parted with their own individuality, and it is the really composite structure which apparently represents a simple and single organism.

Very closely resembling the separate individuals, immediately before the fusion takes place, to form the plasmodium, is a small animacule not unfrequently to be met with in ditches and pools, which is known as an Amœba. The amœba is universally conceded to be of animal affinity, and it compares in many interesting points with the myxomycetes. Like them it exists as a naked mass of protoplasm, which is differentiated into a peripheral clear, and an inner turbid, granular, mass. It also possesses a nucleus and well marked contractile vacuole. The point which interests us just now is the ease with which it encysts itself, on the approach of unfavourable circumstances, only escaping from the cyst, and resuming its activity on the return of suitable conditions of life.

Thus in these simple organisms we see that the formation of cell walls is to be regarded mainly, at any rate, as a means of protection to the living protoplasm· and it scarcely, perhaps, requires insisting on that a permanent ensheathing of the sensitive living substance offers opportunities of development such as could certainly not be utilised by mere naked protoplasm, owing to the disabilities of its physical structure. And, as a matter of fact, we find that in all but these simple or primitive organisms the protoplasm, at least in the adult condition, is enclosed within a membrane or pellicle. It is, however, important to remem, ber that (with but few exceptions) every animal and plant in the earliest embryonic period of its existence repeats the ancestral form, and appears simply as a lump of naked nucleated protoplasm, and that this only subsequently becomes invested by a cuticle, and goes through the more or less complicated development which may happen to be characteristic of the mature structure.

From these early beginnings of differentiation plants and animals proceed on divergent lines, but the essential differences between them are really much less than might easily be imagined. It has already been stated that the membrane is by no means a necessary component of a living body, but nevertheless the special characters which distinguish this membrane when it is present, influence profoundly the further development of the race, for it is just on the difference in the character of their protoplasm envelopes that some of the most important distinctive peculiarities of the two divisions of the organic world depend.

Pleurococcus vulgaris—c, Cell wall; n, Nucleus.

If a typical vegetable cell be compared with a similar one from the animal kingdom, the striking peculiarities of each at once stand out in sharp relief; and moreover, the effect of the respective differences on the general economy of the organism will also be clearly perceived. As an example of an animal cell, we may take a Paramœcium, which belongs to that vast assemblage of unicellular animalcules included in the protozoa. Seen under the microscope, the protoplasm of the paramœcium, besides possessing the usual nucleus, is observed to be invested with a flexible and delicate skin or membrane, which is perforated by numerous holes through which are extruded the well-known whip-like structures called cilia. Locomotion is effected by the rhythmical vibration of these cilia, each of which resembles a flagellum, and, like this body, is simply a protrusion of the cell protoplasm.

At a certain spot on the animal's body there is a depression of the skin, and this depression leads at once to the inner mass of protoplasm of which the animalcule is made up. Currents of water are directed to this aperture by means of cilia, and thus solid particles of food are conveyed through this rudimentary mouth immediately into the protoplasm, where they may be followed, just as in an amœba, during the process of their gradual digestion.

In a typical plant cell, however, the case is widely different. We may take as an example a small unicellular vegetable known as Pleurococcus, which forms the chief constituent of the green incrustation so common upon palings and other woodwork in damp localities. Neglecting for the present the numerous other features of interest which the structure of the pleurococcus offers, we notice at once that the protoplasm is completely enclosed in a membrane, consisting of a substance termed cellulose, of a highly-resistant nature, but which is easily pervious to water and gases, though it will not admit the passage of solid particles. Moreover, in pleurococcus, as in most, though not all, of these lowly plants, since the protoplasm is entirely invested in its cellulose membrane, active movements, like those of paramœcium, are entirely precluded. The vegetable cell, then, as compared with that of an animal, labours under manifest disadvantages both as regards the limited means of acquiring its nutriment, and also as to the form in which it is able to utilise it.

From the unicellular condition it is an easy step to the multicellular; for since all cells are derived from pre-existing ones, by division, a multicellular structure will occur wherever the cells, instead of parting company at once, as isolated individuals, remain in a coherent mass of independent units. And many such cases are known, which may be regarded as the primitive form from which the higher animals and plants have been evolved. As the cellular mass increases in size, certain of the constituents become better fitted, by their position, or in other ways, for the performance of certain functions, and in this way, from a mass of equivalent cells, there arises an organised body.

## Figures and Tables

**Figure f1:**
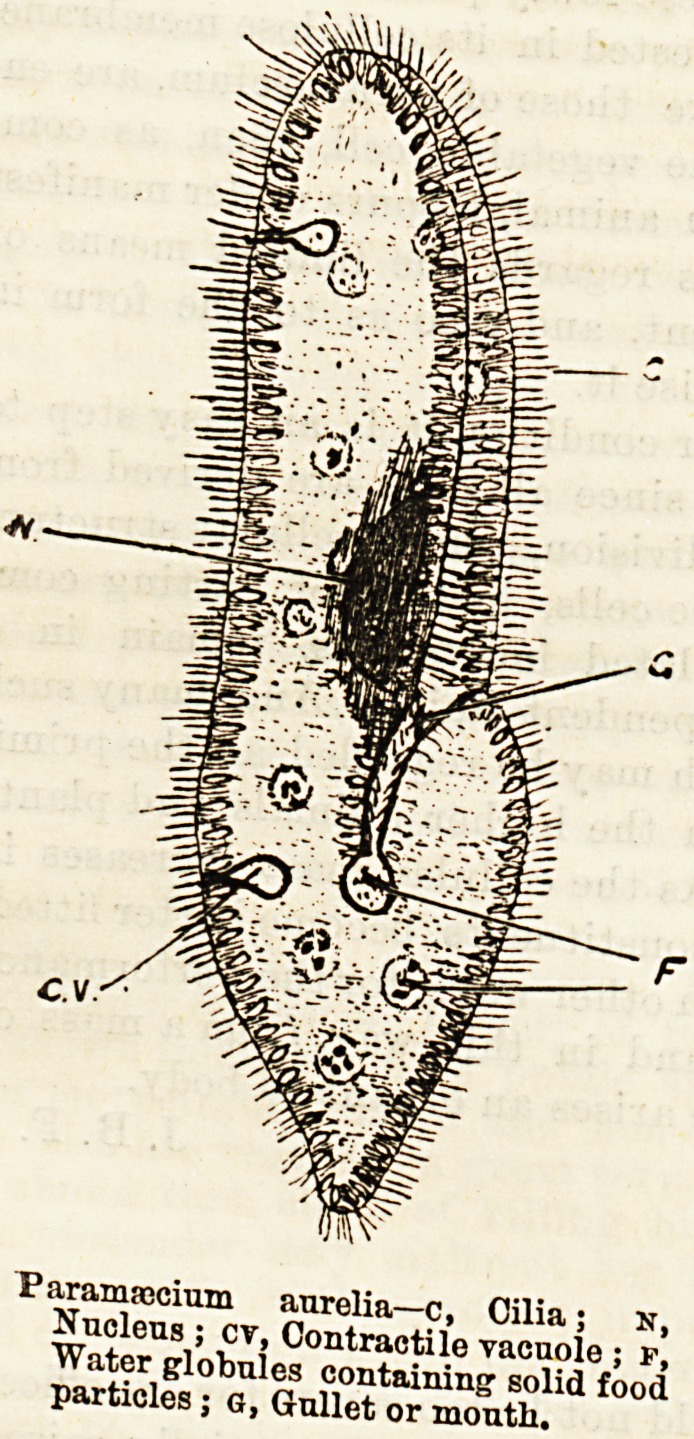


**Figure f2:**